# Effects of Selenocysteine Substitution on the Cyclotide Kalata B2

**DOI:** 10.1002/cbic.70543

**Published:** 2026-09-20

**Authors:** Ramoncito Luis B. de Boda, Sónia Troeira Henriques, Yen‐Hua Huang, Christine C. Hernandez, Portia Mahal G. Sabido, David J. Craik

**Affiliations:** ^1^ Institute for Molecular Bioscience Australian Research Council Centre of Excellence for Innovations in Peptide and Protein Science The University of Queensland Brisbane Queensland Australia; ^2^ Institute of Chemistry College of Science University of the Philippines Diliman Quezon City Philippines; ^3^ School of Biomedical Sciences, Centre for Microbiome Research, and Centre for Immunology and Infectious Control Faculty of Health Translational Research Institute Queensland University of Technology Brisbane Queensland Australia

**Keywords:** cyclotide, kalata B2, NMR spectroscopy, peptides, selenocysteine

## Abstract

Cyclotides are peptides with a head‐to‐tail cyclic backbone and six cysteine residues that are linked in a knotted arrangement. Their unique structure confers exceptional stability and various biological activities, resulting in numerous studies on their synthesis and development for a range of applications. One challenge in cyclotide synthesis is the formation of the correct disulfide bonds that fold the peptide into the native conformation. Replacement of a native disulfide bond with the faster‐forming diselenide bond by substituting cysteine residues with selenocysteine is a strategy that can be implemented to overcome this issue. In this study, we synthesized selenocysteine analogs of the cyclotide kalata B2 and determined the effect of the substitution on folding, structure, and biological activity. The analogs folded faster than their native counterpart, implying that the diselenide bond assists in the formation of the correct structure. HPLC and nuclear magnetic resonance (NMR) analysis revealed a native‐like structure for all analogs. Cytotoxicity and membrane binding assays showed that biological activity was maintained upon selenocysteine substitution. Overall, this study demonstrates the conservative nature of diselenide replacement while improving the folding of kalata B2.

## Graphical abstract

Selenocysteine (Sec) substitution of any of the native complementary cysteine (Cys) residues of the cyclotide kalata B2 preserves the structure, cytotoxicity, and membrane‐binding activity while enhancing its folding efficiency. The study shows the conservative nature and folding benefit of selenocysteine replacement in kalata B2.

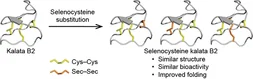

## 1 Introduction

**FIGURE 1:**
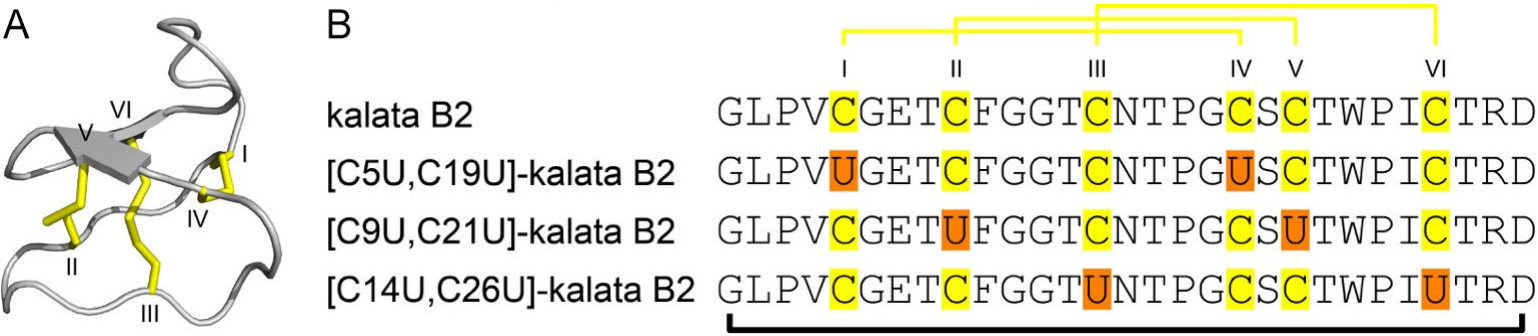
Structure and sequences of cyclotide kalata B2 and its selenocysteine analogs. (A) Solution NMR‐derived three‐dimensional structure of kalata B2 (PDB ID: 1PT4) with cysteine residues labeled using Roman numerals I–VI and disulfide bonds represented by yellow sticks. (B) Amino acid sequences of kalata B2 and selenocysteine analogs synthesized in this study with cysteine and selenocysteine residues highlighted in yellow and orange, respectively, numbered with Roman numerals I–VI, and their connectivities shown in yellow.

Cyclotides are plant‐derived peptides comprising 28–37 amino acids with a head‐to‐tail cyclic backbone and six cysteine residues that are linked in a manner wherein a ring formed by two disulfide bonds and their connecting peptide backbone segments is threaded by a third disulfide bond, resulting in a cyclic cystine knot (CCK) structure (Figure 1) [1, 2]. The unique structure of cyclotides endows them with stability under extreme solution conditions [3] and a diverse spectrum of biological activities that range from pharmaceutical to pesticidal [4]. These attributes make cyclotides interesting peptides to synthesize and develop for research and commercial applications.

The chemical synthesis of cyclotides is typically achieved by assembling the linear peptide chain, cyclizing it, and oxidizing the disulfide bonds to fold the peptide into the CCK structure [5]. The last step can be considered the most difficult due to the possibility of forming one of the 14 isomers with non‐native cross‐links as a consequence of having six cysteine residues. It is therefore important to determine a method that would yield more of the correctly folded cyclotide and less of the misfolded isomers. Initial reports on cyclotide synthesis achieved the native structure via a two‐step disulfide bond formation that involves the stepwise deprotection and oxidation of two sets of cysteine residues with orthogonal thiol‐protecting groups [6, ‍7]. Another study on the synthesis of the prototypical cyclotide kalata B1 presented a one‐step approach by using an alkaline buffer containing a disulfide shuffling reagent to aid in forming the correct cystine pairing and an organic cosolvent to stabilize the hydrophobic residues found on the surface of kalata B1 [8]. The latter method does not require different sets of thiol protecting groups, thus simplifying the folding step, but folding reagents and conditions vary for each cyclotide, which makes it challenging to determine the appropriate condition that would facilitate efficient folding [9, 10, 11, 12]. Sequence modification has also been used to enable the folding of a cyclotide under conditions wherein the native peptide does not correctly fold in vitro [13].

Replacement of a disulfide bond with a diselenide bond via selenocysteine (Sec, U) substitution of complementary cysteine residues is a strategy that can be used to overcome this hurdle in oxidative folding. Selenocysteine is analogous to cysteine—with only a selenol group in place of a thiol group as a side chain—and dimerizes upon oxidation to form a structure similar to cystine; thus, substituting cysteine with selenocysteine can be regarded as highly conservative [14]. Despite their resemblance, selenocysteine dimerizes faster than cysteine due to the lower p*K*_a_ of the selenol group (5.24) than that of the thiol group (8.25), with the former existing predominantly as a reactive selenolate ion, while the latter remaining primarily protonated and uncharged at normal cell and body pH (7.4) [15]. Moreover, the lower redox potential of a diselenide bond (−381 mV) suggests that it is more thermodynamically stable than a disulfide (−180 mV) or a selenosulfide bond (−326 mV) [16]. The regioselective formation of a diselenide bond can therefore aid disulfide‐rich peptides in folding into the correct structure. Selenocysteine substitution has been applied to disulfide‐containing peptides and proteins such as endothelin‐1 [17], apamin [18, 19], conotoxins [20, 21, 22, 23, 24], bovine pancreatic trypsin inhibitor [25], insulin [26, 27, 28], hirudin [29], epidermal growth factor [30], and relaxin‐2 [31] with favorable results: the selenocysteine analogs folded more easily, their structure was nearly identical to the native structure, and their activity was either retained or improved.

In the current study, we applied the selenocysteine substitution strategy to kalata B2 (Figure 1), a cyclotide isolated from *Oldenlandia affinis* with a structure similar to kalata B1 [32] but reported to have improved membrane‐binding [33] and cytotoxicity towards cancer cells [34]. We investigated the effects of diselenide replacement of each native disulfide bridge on the peptide’s folding, structure, membrane binding, and lung cancer cell toxicity.

## 2 Results and Discussion

### 2.1 Synthesis of Selenocysteine Kalata B2 Analogs

The selenocysteine kalata B2 analogs synthesized in this study, namely, [C5U,C19U]‐kalata B2, [C9U,C21U]‐kalata B2, and [C14U,C26U]‐kalata B2, have a single diselenide bond in place of a native disulfide bond for each connectivity (Figure 1B). Analogs were assembled using Fmoc solid‐phase peptide synthesis, and selenocysteine residues were incorporated with the 4‐methoxybenzyl group protecting the side chain. After resin cleavage, backbone cyclization, and side chain deprotection, the cyclic, reduced precursors were obtained with a mass of 3053.2 Da, which is 2 Da less than the predicted mass (3055.2), suggesting the presence of a diselenide bond in all the peptides. The formation of a diselenide bridge prior to folding has also been observed in the synthesis of selenocysteine analogs of multiple conotoxins [21, 22, 23, 24].

Oxidative folding of the analogs was carried out in a buffer that was reported to efficiently fold kalata B2, composed of equal parts of 2‐propanol and basic NH_4_HCO_3_ solution, with equimolar concentrations of reduced (GSH) and oxidized glutathione (GSSG) [10]. The alkaline buffer ionizes the thiol group of the cysteine residues, making it more reactive and easier to oxidize into a disulfide, while 2‐propanol assists in the correct folding of the cyclotides by stabilizing the hydrophobic residues that are surface‐exposed in the native structure. GSSG and GSH aid in the folding process, with the former acting as an oxidant and the latter allowing disulfide interchange when a non‐native disulfide bridge is formed.

**FIGURE 2:**
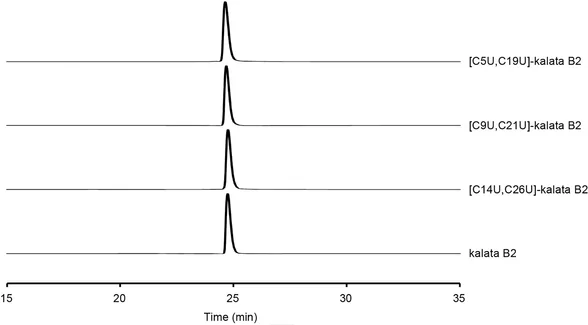
LC traces of selenocysteine kalata B2 analogs compared to kalata B2.

Upon completion of the folding step, the analogs were purified using RP‐HPLC and characterized using LC–MS. The LC traces in Figure 2 show that retention time of the analogs is similar to that of kalata B2, which implies that replacement of a disulfide bond with a relatively hydrophobic diselenide bond does not affect the overall hydrophobicity of the cyclotide as a result of the CCK motif, wherein the disulfide bonds are buried within the peptide core [32]. The observed masses of the analogs were identical to the expected mass of 3049.2 Da (Figures S1–S3), confirming the synthesis of these peptides. Similar LC elution profiles were also observed for the CCK‐containing kalata B1 and its selenocysteine analog (Figure S4).

### 2.2 Structural Analysis by 2D Nuclear Magnetic Resonance (NMR) Spectroscopy

**FIGURE 3:**
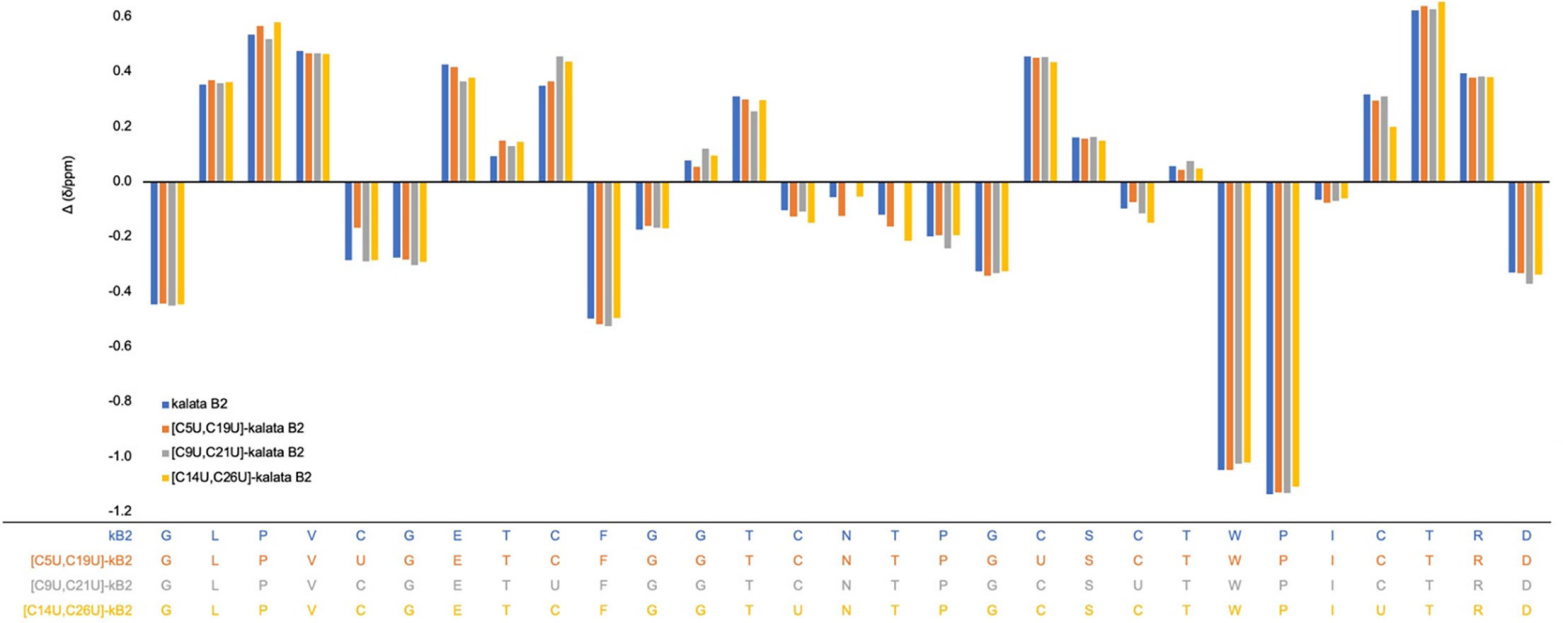
Secondary αH NMR chemical shift comparison of selenocysteine kalata B2 analogs and kalata B2. Peptide sequences are shown at the bottom.

We compared the structure of the analogs with kalata B2 using secondary αH NMR chemical shifts derived from 2D TOCSY and NOESY spectra. Secondary chemical shifts are widely used markers of peptide structure, calculated as the difference between the NMR αH chemical shift of a given residue in a peptide and the corresponding αH chemical shift of that residue in a random coil peptide. The secondary αH chemical shift pattern of the analogs is similar to that of kalata B2, with a difference of less than 0.2 ppm in the secondary αH chemical shifts of all residues (Figure 3). The αH peaks of the Asn15 and Thr16 residues of [C9U,C21U]‐kalata B2 did not appear in the TOCSY and NOESY spectra, and thus, their secondary chemical shifts could not be determined. While the reason for their absence is not clear, it is likely that these peaks are broader and thus of lower intensity than other peaks. Nevertheless, the secondary αH shift similarity of the analogs with kalata B2 indicates that their structure exhibits a native‐like conformation.

### 2.3 Oxidative Refolding Monitoring

**FIGURE 4:**
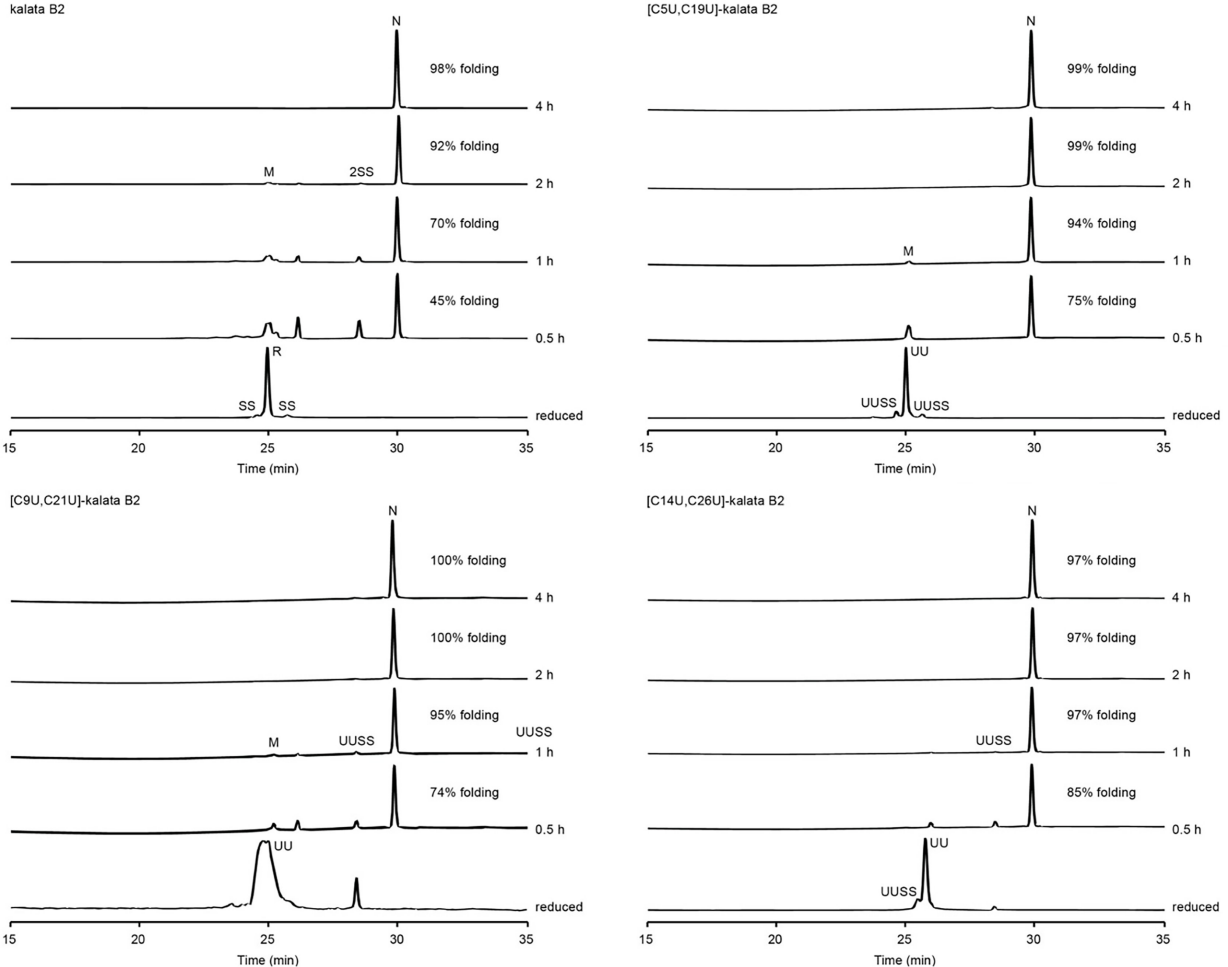
LC traces of the reduced, cyclic kalata B2 and selenocysteine analogs and their oxidative refolding. Oxidation of 30 µM peptides was performed in 1:1 (v/v) 2‐propanol/0.1 M NH_4_HCO_3_ (pH 8.5) with 2 mM/2 mM GSH/GSSG at room temperature. Aliquots were taken at different time points, quenched by adding TFA to a concentration of 0.5%, and analyzed using LC–MS. Time points are labeled on the LC traces. The peaks corresponding to the reduced peptide (R), one‐disulfide species (SS), two‐disulfide species (2SS), diselenide species (UU), diselenide and disulfide species (UUSS), misfolded (M), and native or native‐like peptide (N) are labeled. Accumulation (%) of the natively folded peptide are shown on the LC traces.

To evaluate the folding of the selenocysteine analogs in comparison with kalata B2, we first reduced the peptides with dithiothreitol (DTT) and purified the reduced peptides using RP‐HPLC. Oxidative refolding was performed on the cyclic, reduced peptides using the same folding buffer at a concentration of 30 µM. Aliquots were taken out at selected time points, diluted with an aqueous TFA solution to stop the reaction, and analyzed using LC–MS. Folding yield was determined by calculating the fraction of the natively folded peptide relative to the total detectable species at each time point. As presented in Figure 4, the one‐disulfide species (SS) was already observed on reduced kalata B2 after dissolving it in water, while the reduced analogs consist mostly of the diselenide‐containing species (UU), further demonstrating the stability of the diselenide bond in reducing conditions. In addition, species containing both diselenide and disulfide bonds (UUSS) already appear in the reduced analogs, implying their rapid formation. Based on the quenching time points, the analogs were completely folded after 2 h, while kalata B2 was completely folded only after 4 h. This is probably due to the presence of a preformed diselenide bond with a native connectivity, which greatly reduces the potential for alternative pathways to be accessed in the folding landscape, allowing the analogs to easily fold into the correct conformation. Furthermore, a diselenide bond, once formed, is less likely to shuffle compared to disulfide or selenosulfide bonds, thereby locking a native connectivity early in the folding pathway. [C14U,C26U]‐Kalata B2, in which the Cys^III^–Cys^VI^ connectivity is replaced with a diselenide bond, exhibits the highest folding yield of CCK‐folded peptide among the selenocysteine analogs after 30 min and 1 h; moreover, it did not show any peak corresponding to a non‐native folded species (M), unlike the other analogs. These results suggest that forming the Cys^III^–Cys^VI^ connectivity first is beneficial for the folding of kalata B2 as it minimizes the production of misfolded isomers.

### 2.4 Biological Activity

**FIGURE 5:**
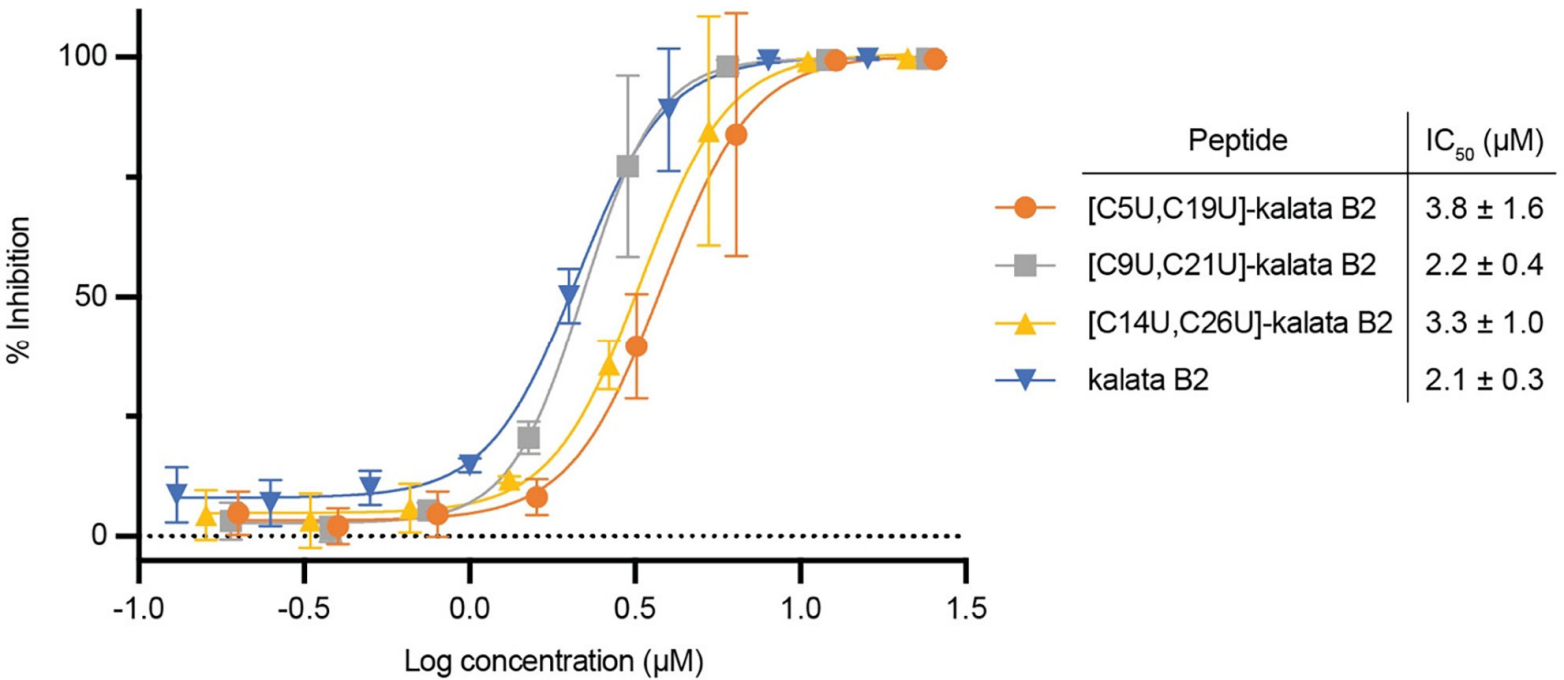
Cytotoxic dose–response curve and IC_50_ values of selenocysteine kalata B2 analogs and kalata B2 against A549 cells. Varying concentrations of the peptides were incubated with the cells at 37 °C and 5% CO_2_ for 1 h and the percentage of growth inhibition determined using a resazurin‐based assay. IC_50_ values represent the mean ± standard deviation (*N* = 3).

To assess the effect of diselenide replacement on the biological activity of kalata B2, we tested the cytotoxicity of the analogs on human lung cancer (A549) cells and their binding to a synthetic membrane. Cell viability after treatment of the peptide with different concentrations was measured using the fluorometric resazurin assay with water and Triton X‐100 as the negative (0%) and positive (100%) controls, respectively. IC_50_ values were then determined from the dose–response curve. As shown in Figure 5, the analogs exhibit A549 toxicity comparable to kalata B2.

**FIGURE 6:**
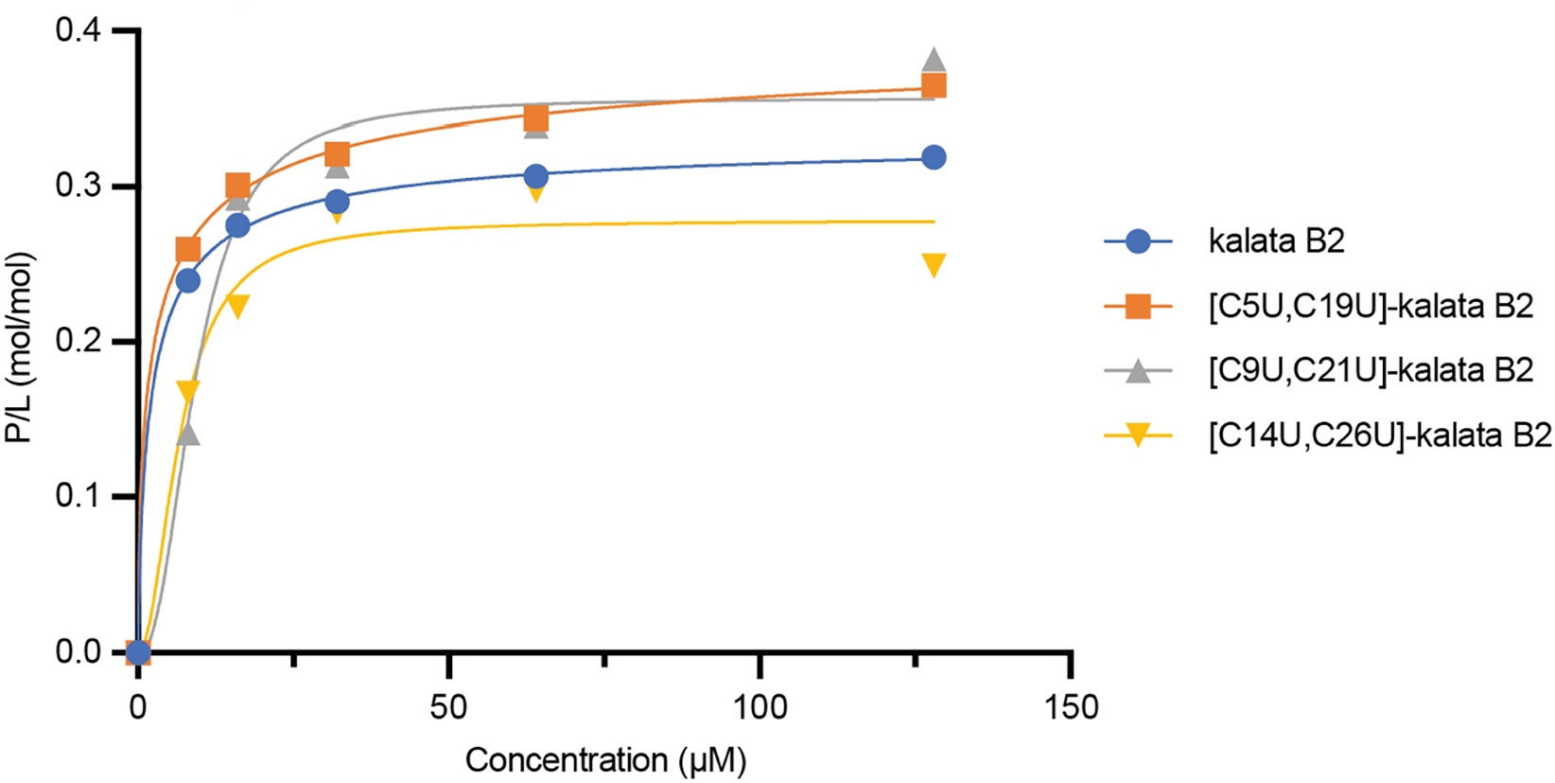
Binding dose–response curve of selenocysteine kalata B2 analogs and kalata B2 over POPC/POPE (80:20) bilayers onto L1 chip surface monitored by SPR.

Kalata B2 is reported to effectively bind to membranes containing phospholipids with phosphatidylethanolamine (PE) headgroups via a distinct patch on its surface called the bioactive face [33]. To determine if the binding affinity to PE is maintained upon diselenide replacement, binding of the analogs on a model membrane composed of palmitoyloleoylphosphatidylcholine (POPC) and palmitoyloleoylphosphatidylethanolamine (POPE) with a molar ratio of 80:20 was examined using surface plasmon resonance (SPR). The peptide‐to‐lipid molar ratio (P/L) was calculated and plotted against the peptide concentration injected to produce the dose–response curve. As shown in Figure 6, small differences in the membrane binding curves of the analogs and kalata B2 can be observed, implying that there is minimal perturbation of the bioactive face due to the preservation of the native fold. PE phospholipids are present in high concentrations in the outer membrane of cancer cells [35]; thus, the similarity of the peptides’ membrane‐binding activity accounts for the similarity in their cytotoxicity. PE binding was also observed on the selenocysteine analog of kalata B1 (Figure S2).

## 3 Conclusion

In summary, diselenide replacement of any of the native disulfide bonds of kalata B2 enhanced the peptide’s folding, preserved the native fold, and retained its activity against human lung cancer cells and its binding toward PE‐rich membranes, making this modification on the cyclotide conservative as far as structure and activity are concerned but more efficient in terms of folding. This strategy was also successfully implemented on kalata B1 (Supporting Information), which has a similar structure to kalata B2, thus pointing to the more general potential of selenocysteine replacement in Möbius cyclotides [1, 2]. More generally, the findings suggest that selenocysteine substitution is a useful strategy for cyclotides that are difficult to fold in vitro and that it could be useful to aid our understanding of the folding mechanism of disulfide‐rich cyclic peptides. Beyond facilitating folding, selenocysteine‐containing cyclotides could potentially act as reservoirs for selenium sequestration or biofortification by bioengineering plants to produce these peptides, although the toxicity of these peptides should also be taken into account, considering the narrow range of selenium concentrations between micronutrient and toxin [36].

## 4 Experimental Section

### 4.1 Synthesis of Selenocysteine Kalata B2 Analogs

Peptides were synthesized according to the method described by Cheneval et al. [37]. Peptide chains were assembled by Fmoc solid‐phase chemistry on an automated synthesizer (Symphony, Gyros Protein Technologies) on a scale of 0.125 mmol per peptide using 2‐chlorotrityl chloride resin as a solid support. Side‐chain protecting groups used were the following: Cys(Trt), Asp(tBu), Glu(tBu), Asn(Trt), Arg(Pbf), Ser(tBu), Thr(tBu), Sec(Mob), and Trp(Boc). Side‐chain‐protected peptide precursors were cleaved from the resin using 1% (v/v) TFA in DCM and were C‐to‐N cyclized using two molar equivalents of hexafluorophosphate azabenzotriazole tetramethyl uronium (HATU) and five molar equivalents of *N*,*N*‐diisopropylethylamine (DIPEA) in DMF for 3 h. Precursors were treated with 95:2.5:2.5 (v/v/v) TFA/triisopropylsilane/water to remove the side chain protecting groups. Crude cyclic, reduced peptides were purified in a RP‐HPLC system (Shimadzu LC‐20AD) using a preparative C18 column (Phenomenex) on a gradient of solvent B (0.05% TFA, 90% (v/v) acetonitrile in water) in solvent A (0.05% (v/v) TFA in water). Purified cyclic reduced peptides with the following weights ([C5U,C19U]‐kalata B2: 30 mg; [C9U,C21U]‐kalata B2: 10 mg; and [C14U,C26U]‐kalata B2: 40 mg) were folded in a buffer described by Aboye et al. [10] Peptides were dissolved in 1:1 (v/v) 2‐propanol/0.1M NH_4_HCO_3_ (pH 8.5) mixture with 2 mM GSH and GSSG to a final concentration of 0.5 mg/mL and stirred for 24 h at room temperature. TFA was added to the mixture at a concentration of 0.1% to stop the reaction. Folded peptides were purified using RP‐HPLC and analyzed using LC–MS. Yields and observed masses of the analogs (calculated mass: 3049.2 Da) are the following: [C5U,C19U]‐kalata B2: 1.35 mg (0.4%), 3049.2 Da; [C9U,C21U]‐kalata B2: 0.51 mg (0.1%), 3049.2 Da; [C14U,C26U]‐kalata B2: 1.10 mg (0.3%), 3049.2 Da.

### 4.2 NMR Analysis

Pure peptides were dissolved in 30% CD_3_CN in water with 0.002 mg 4,4‐dimethyl‐4‐silapentane‐1‐sulfonic acid (DSS) for chemical shift reference. Total correlation spectroscopy (TOCSY) and nuclear Overhauser effect spectroscopy (NOESY) spectra were acquired at 298 K with a 600 MHz NMR spectrometer (Bruker ARX). Spectral assignments were done using CcpNmr Analysis (version 2.4.2) [38]. Secondary αH chemical shifts were determined by calculating the difference between the observed αH chemical shift of each residue and its corresponding random coil αH chemical shift [39]. For Sec residues, the random coil value of Cys was subtracted from the observed shift values.

### 4.3 Reductive Unfolding and Oxidative Refolding Monitoring

Kalata B2 and analogs were dissolved in 0.1 M NH_4_HCO_3_ (pH 8.0) with 5 mM DTT and stirred for 2 h at room temperature. Reduced peptides were purified using RP‐HPLC. Oxidative refolding was performed on reduced peptides with a concentration of 30 µM in the same folding buffer (1:1 (v/v) 2‐propanol/0.1M NH_4_HCO_3_ (pH 8.5) with 2 mM GSH and GSSG) at room temperature. To monitor the folding reaction, aliquots were removed at 0.5, 1, 2, and 4 h, diluted with 0.5% TFA, and analyzed using LC–MS. Reduced peptides dissolved in water and diluted in 1:1 water/acetonitrile were also analyzed using LC–MS (Figures S5–S8). The accumulation of the natively folded peptide at a given time point was calculated by integrating the LC trace.

### 4.4 Cell Culture

Human epithelial lung cancer cells A549 were cultured in Ham’s F‐12K medium supplemented with 1% (v/v) penicillin/streptomycin and 10% (v/v) FBS and were grown in a humidified incubator under standard culture conditions (5% CO_2_ at 37 °C and 95% humidity).

### 4.5 Cell Viability Assay

Cells were seeded the day before the assay in 96‐well tissue culture‐treated plates (1 × 10^4^ cells/well). Peptides were dissolved and diluted in sterile water and were further diluted tenfold with serum‐free medium and were tested in triplicate. Peptide solutions were then incubated together with the cells for 1 h. Water and 0.01% (v/v) Triton‐X were included as controls, indicating 100% and 0% cell viability, respectively. Following incubation, peptide solutions were removed, and aliquots of 10 µL of 0.05% (w/v) sterile resazurin were added to each well containing 100 µL of fresh medium. Cells were incubated under standard conditions of 37 °C and 5% CO_2_ overnight. The fluorescence of the plates was measured on a plate reader at 560 nm excitation and 585 nm emission wavelengths.

### 4.6 Preparation of Lipid Vesicles

Synthetic lipids POPC and POPE were obtained from Avanti Polar Lipids. Small unilamellar vesicles (SUVs, 50 nm diameter) of POPC/POPE (80:20) were prepared by freeze‐thaw fracturing and size extrusion, as described previously [33]. Vesicle suspensions were prepared in HEPES (10 mM, pH 7.4) containing NaCl (150 mM).

### 4.7 Peptide–Membrane Interaction Monitoring

Membrane binding of peptides tested to POPC/POPE (80:20) bilayers was determined using SPR at 25 ºC, as described previously [33].

## Funding

This work was supported by the University of the Philippines Office of the Vice President for Academic Affairs Emerging Interdisciplinary Research Program (EIDR‐C06‐021), Department of Science and Technology, University of the Philippines Office of International Linkages COOPERATE Program, National Health and Medical Research Council (GNT 2009564), Australian Research Council Centre of Excellence for Innovations in Peptide and Protein Science (CE200100012).

## Conflicts of Interest

The authors declare no conflicts of interest.

## Supporting information

Supplementary Material

## Data Availability

The data that supports the findings of this study are available in the supplementary material of this article.
