# Supplementary material for: Effects of Selenocysteine Substitution on the Cyclotide Kalata B2
Source: Chembiochem. 2026 Sep 20;27(18):e70543. doi: 10.1002/cbic.70543 (PMC13591204; doi:10.1002/cbic.70543)
Supplement: Supplementary file 1 [file CBIC-27-e70543-s001.pdf]

Supporting Information

Effects of Selenocysteine Substitution on the Cyclotide Kalata B2

Ramoncito Luis B. de Boda,[a,b] Sónia Troeira Henriques,[a,c] Yen-Hua Huang,[a] Christine C. Hernandez,[b] Portia Mahal G. Sabido,*[b] and David J. Craik*[a]

[a] Mr. R. L. B. de Boda, Assoc. Prof. S. T. Henriques, Dr. Y.-H. Huang, Prof. D. J. Craik
Institute for Molecular Bioscience
Australian Research Council Centre of Excellence for Innovations in Peptide and Protein Science
The University of Queensland
Brisbane 4072 Queensland, Australia
E-mail: d.craik@imb.uq.edu.au

[b] Mr. R. L. B. de Boda, Prof. C. C. Hernandez, Dr. P. M. G. Sabido
Institute of Chemistry
College of Science
University of the Philippines Diliman
Quezon City 1101, Philippines
E-mail: pgsabido@upd.edu.ph

[c] Assoc. Prof. S. T. Henriques
School of Biomedical Sciences
Faculty of Health
Translational Research Institute
Queensland University of Technology
Brisbane 4102 Queensland, Australia

Table of Contents

Figure S1. Chromatogram and mass spectrum of crude linear, reduced [C5U,C19U]-kalata B2 precursor. (A) LC trace of the crude product with the peak corresponding to the peptide precursor marked with an asterisk. (B) ESI-MS spectrum of the marked peak.

Figure S2. Chromatogram and mass spectrum of crude linear, reduced [C9U,C21U]-kalata B2 precursor. (A) LC trace of the crude product with the peak corresponding to the peptide precursor marked with an asterisk. (B) ESI-MS spectrum of the marked peak.

Figure S3. Chromatogram and mass spectrum of crude linear, reduced [C14U,C26U]-kalata B2 precursor. (A) LC trace of the crude product with the peak corresponding to the peptide precursor marked with an asterisk. (B) ESI-MS spectrum of the marked peak.

Figure S4. ESI-MS spectrum of pure [C5U,C19U]-kalata B2.

Figure S5. ESI-MS spectrum of pure [C9U,C21U]-kalata B2.

Figure S6. ESI-MS spectrum of pure [C14U,C26U]-kalata B2.

Figure S7. LC traces of pure [C5U,C19U]-kalata B1 compared to kalata B1.

Figure S8. ESI-MS spectrum of cyclic, reduced kalata B2.

Figure S9. ESI-MS spectrum of cyclic, reduced [C5U,C19U]-kalata B2.

Figure S10. ESI-MS spectrum of cyclic, reduced [C9U,C21U]-kalata B2.

Figure S11. ESI-MS spectrum of cyclic, reduced [C14U,C26U]-kalata B2.

Figure S12. Binding dose-response curve of kalata B1 and [C5U,C19U]-kalata B1 over POPC/POPE (80:20) bilayers onto L1 chip surface.

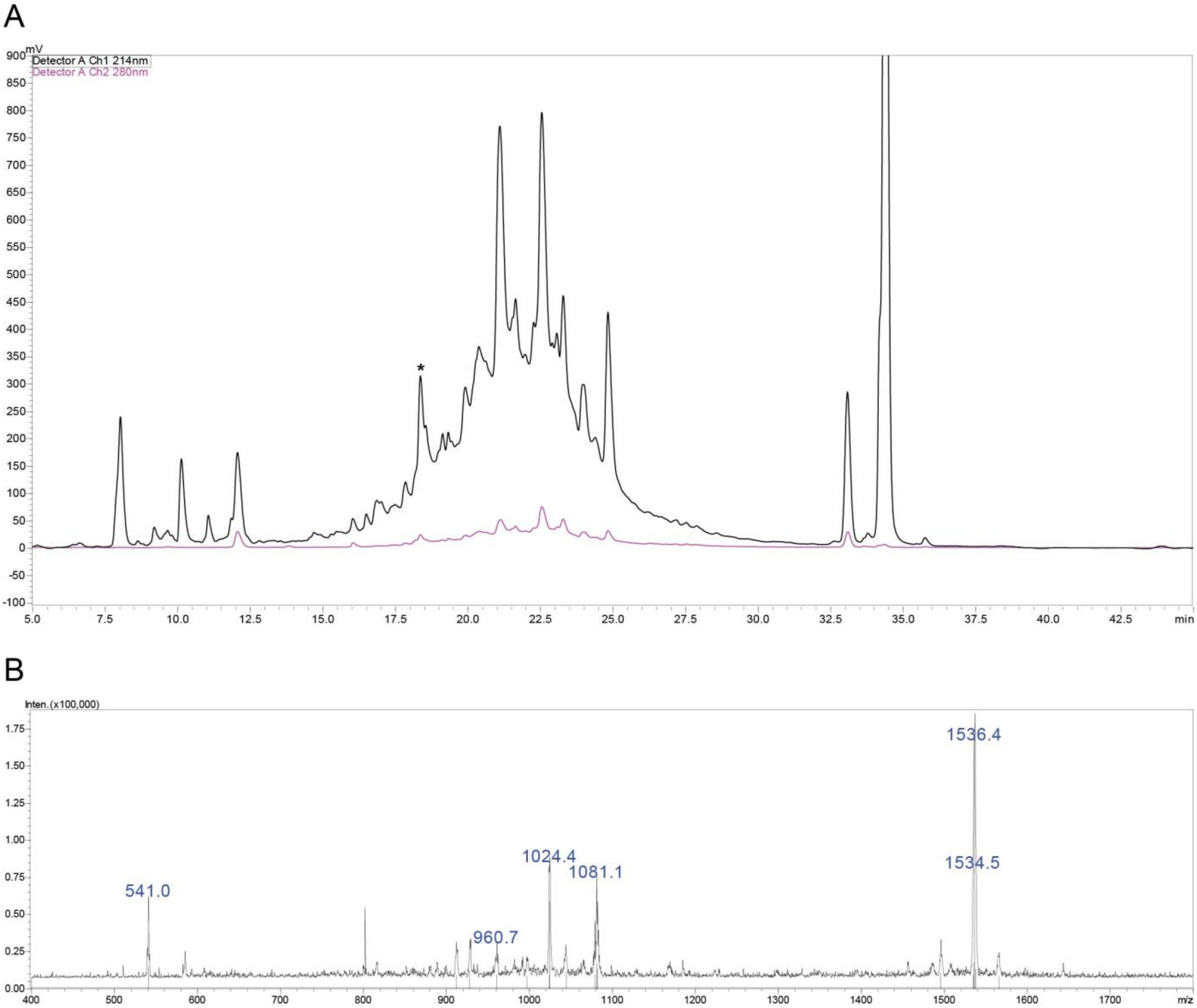

Figure S1. Chromatogram and mass spectrum of crude linear, reduced [C5U,C19U]-kalata B2 precursor. (A) LC trace of the crude product with the peak corresponding to the peptide precursor marked with an asterisk. (B) ESI-MS spectrum of the marked peak.

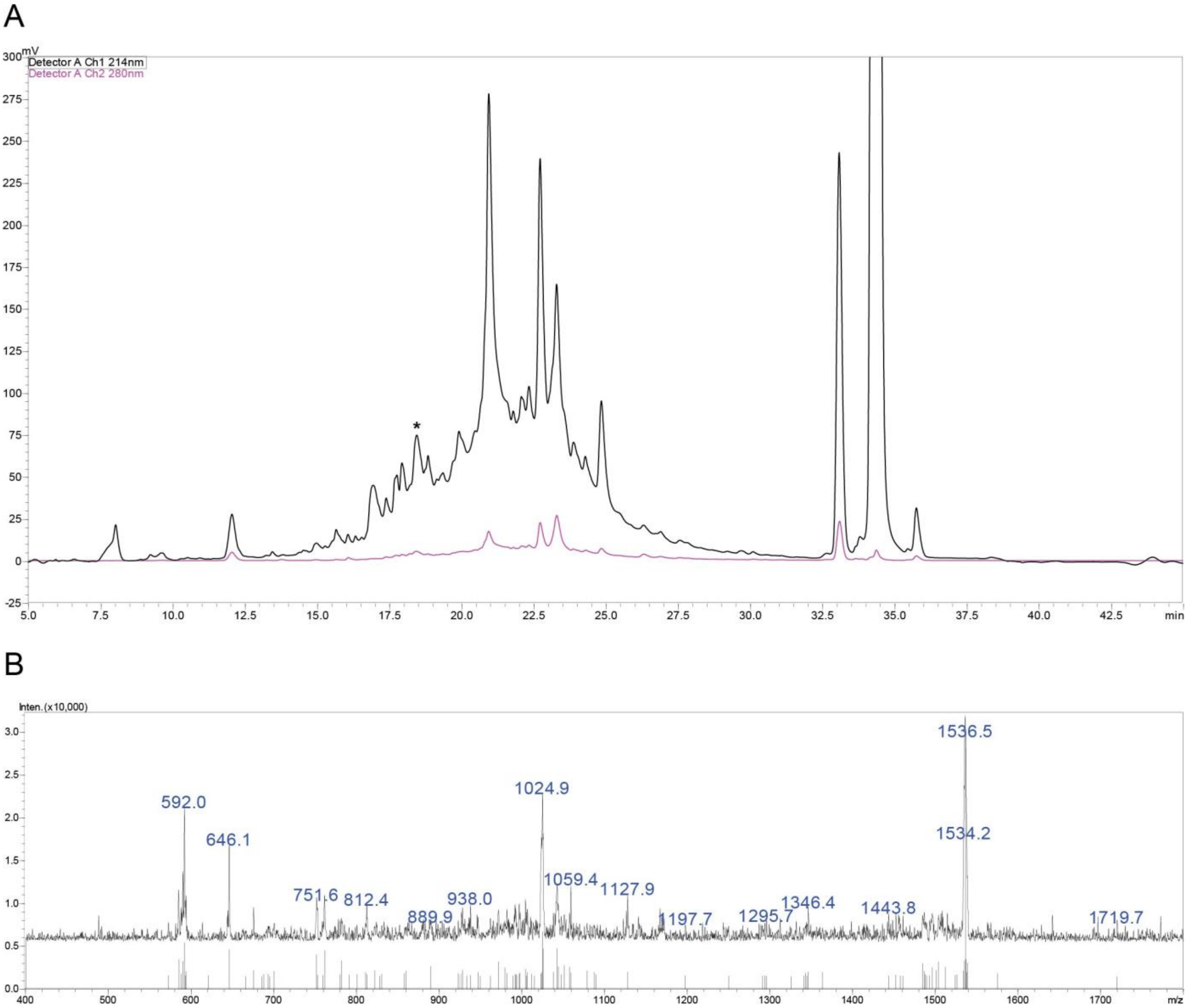

Figure S2. Chromatogram and mass spectrum of crude linear, reduced [C9U,C21U]-kalata B2 precursor. (A) LC trace of the crude product with the peak corresponding to the peptide precursor marked with an asterisk. (B) ESI-MS spectrum of the marked peak.

A

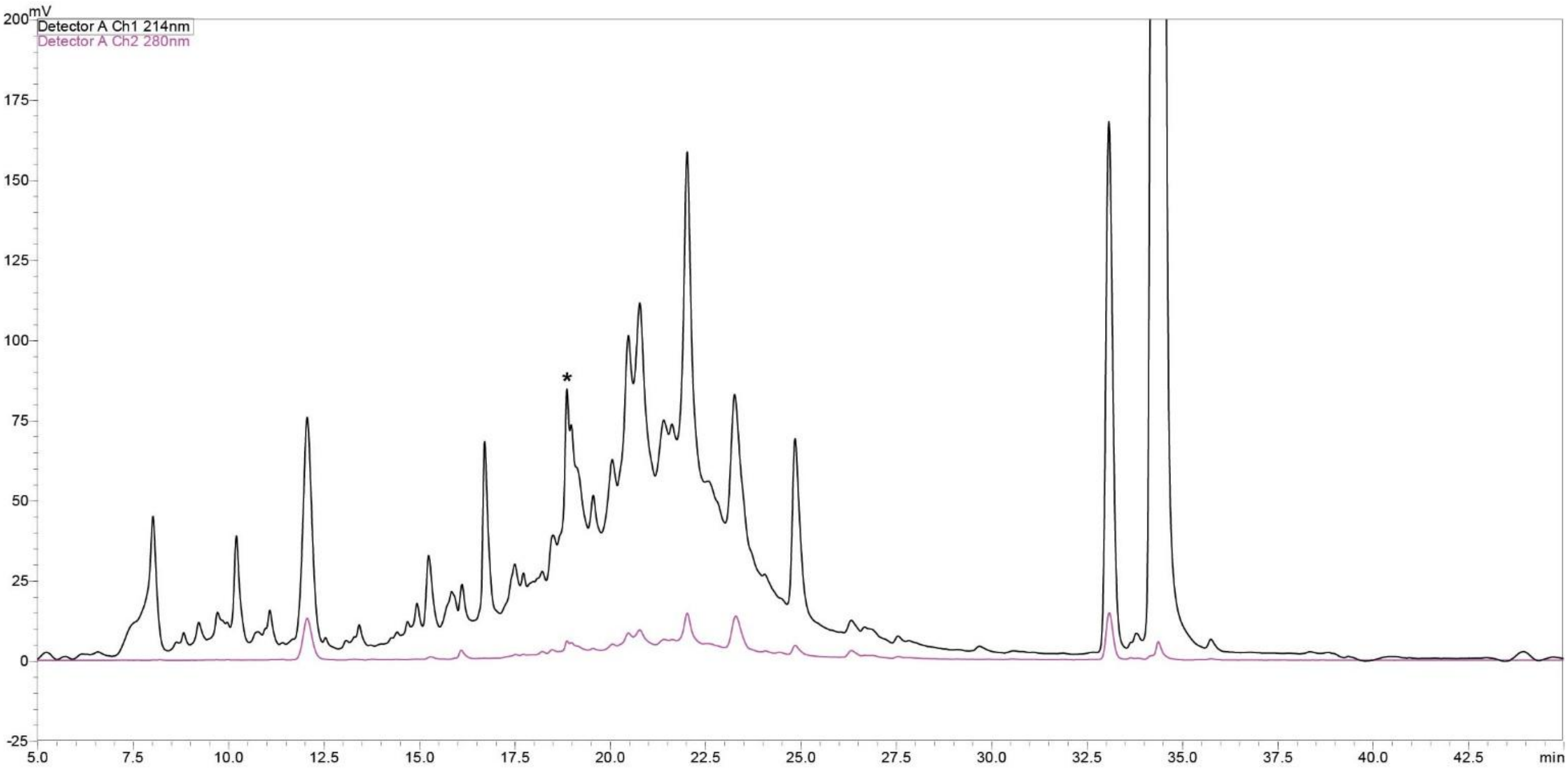

B

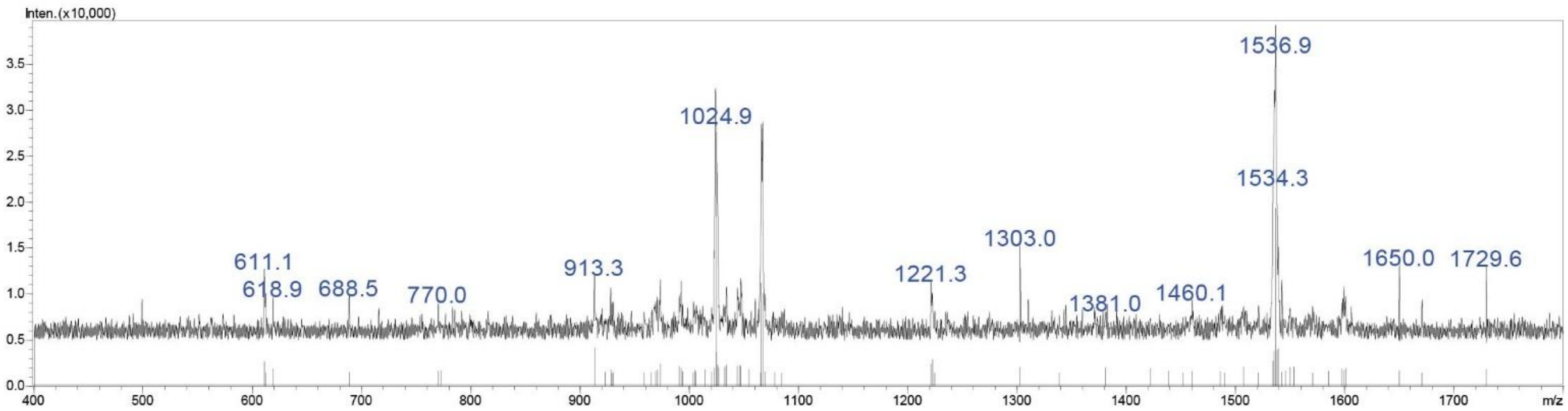

Figure S3. Chromatogram and mass spectrum of crude linear, reduced [C14U,C26U]-kalata B2 precursor. (A) LC trace of the crude product with the peak corresponding to the peptide precursor marked with an asterisk. (B) ESI-MS spectrum of the marked peak.

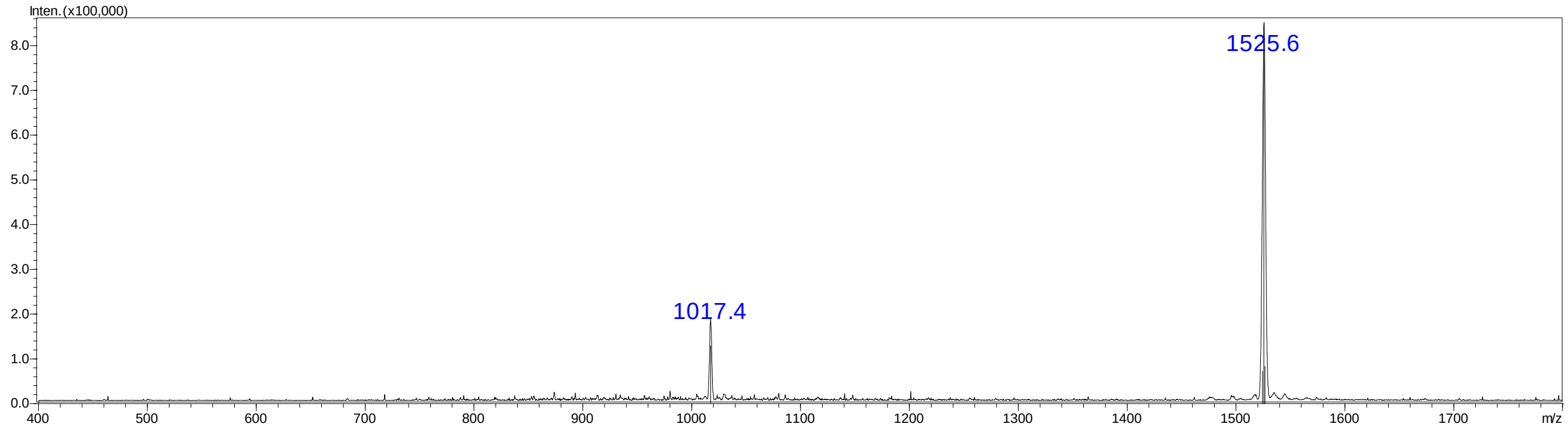

Figure S4. ESI-MS spectrum of pure [C5U,C19U]-kalata B2.

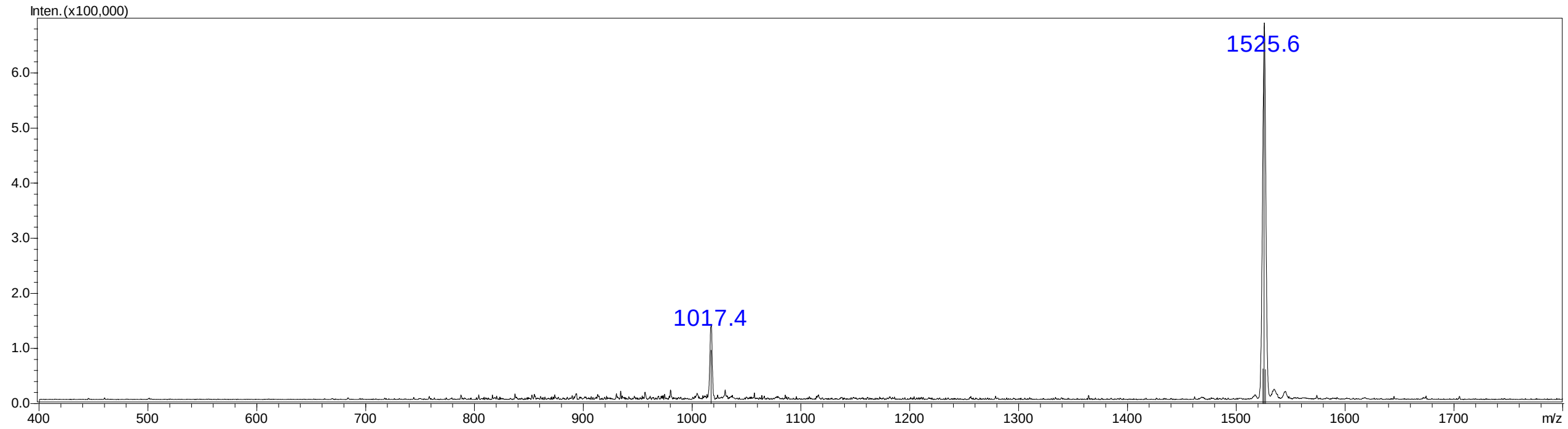

Figure S5. ESI-MS spectrum of pure [C9U,C21U]-kalata B2.

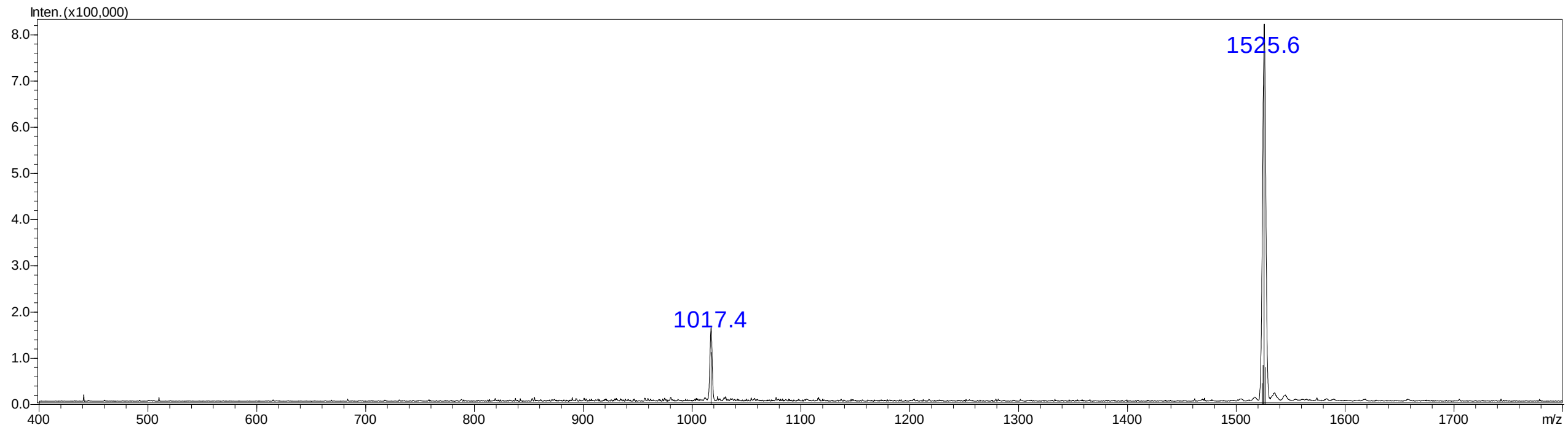

Figure S6. ESI-MS spectrum of pure [C14U,C26U]-kalata B2.

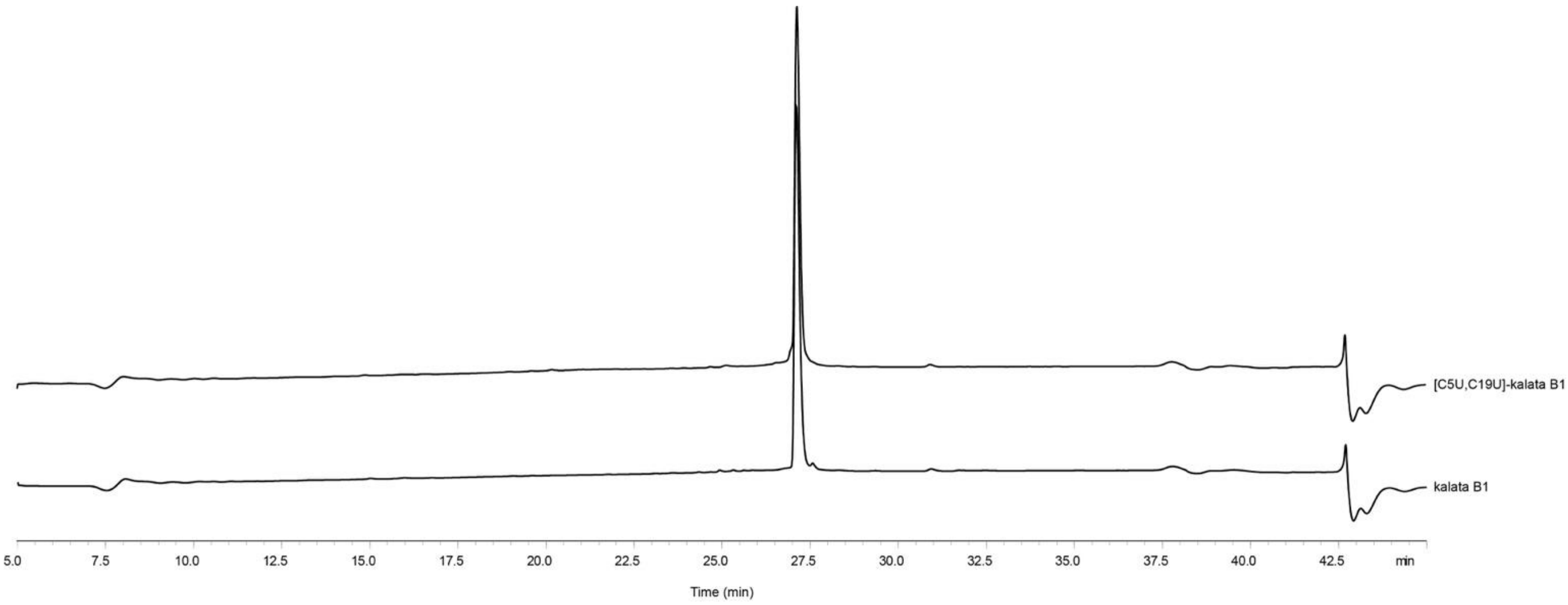

Figure S7. LC traces of pure [C5U,C19U]-kalata B1 compared to kalata B1.

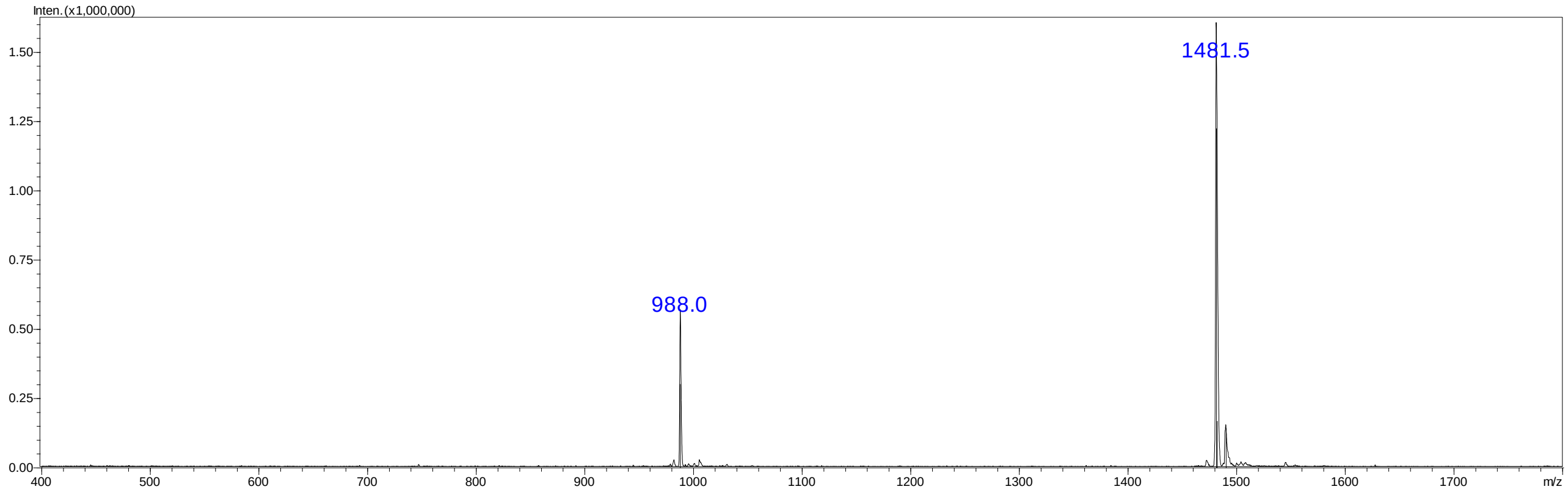

Figure S8. ESI-MS spectrum of cyclic, reduced kalata B2.

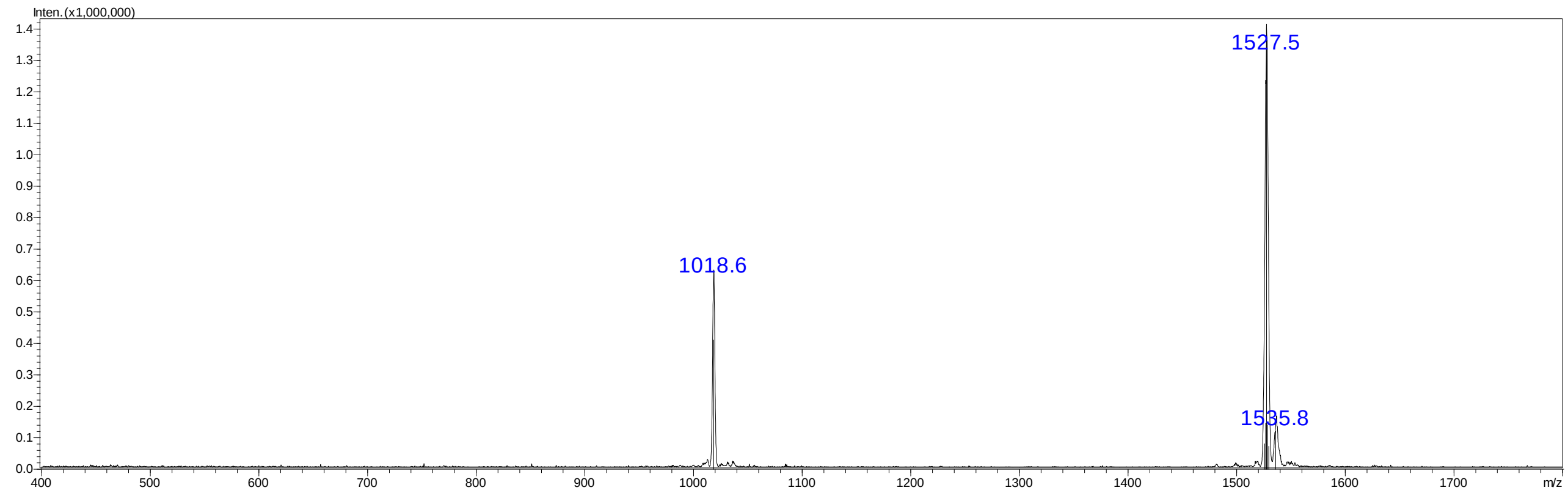

Figure S9. ESI-MS spectrum of cyclic, reduced [C5U,C19U]-kalata B2.

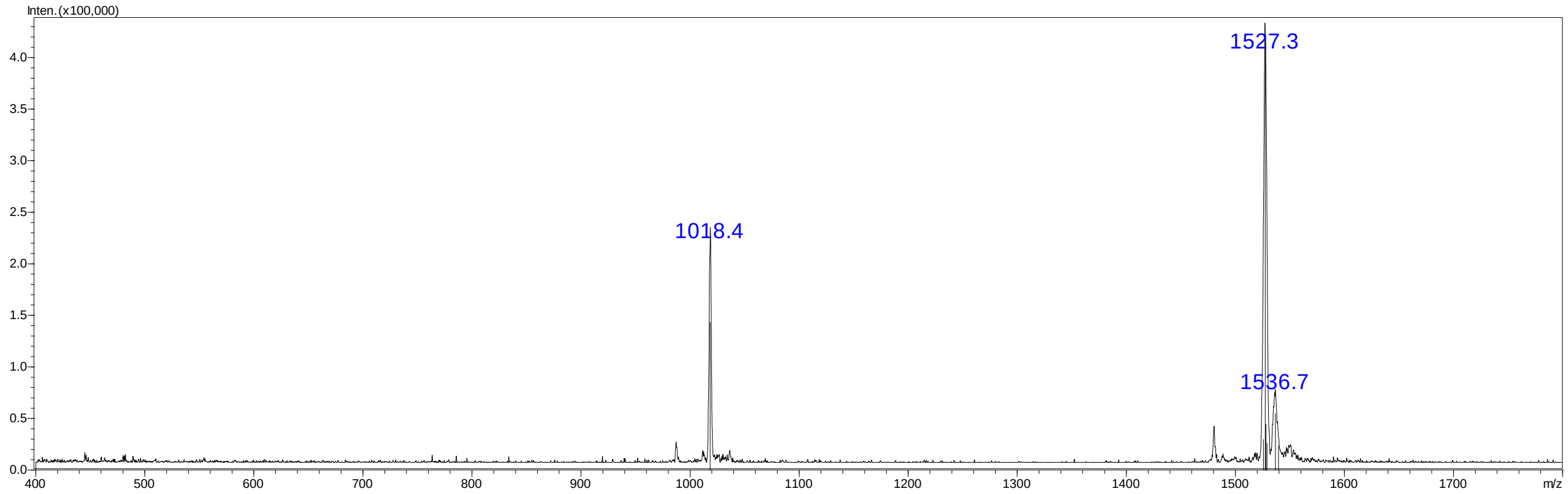

Figure S10. ESI-MS spectrum of cyclic, reduced [C9U,C21U]-kalata B2.

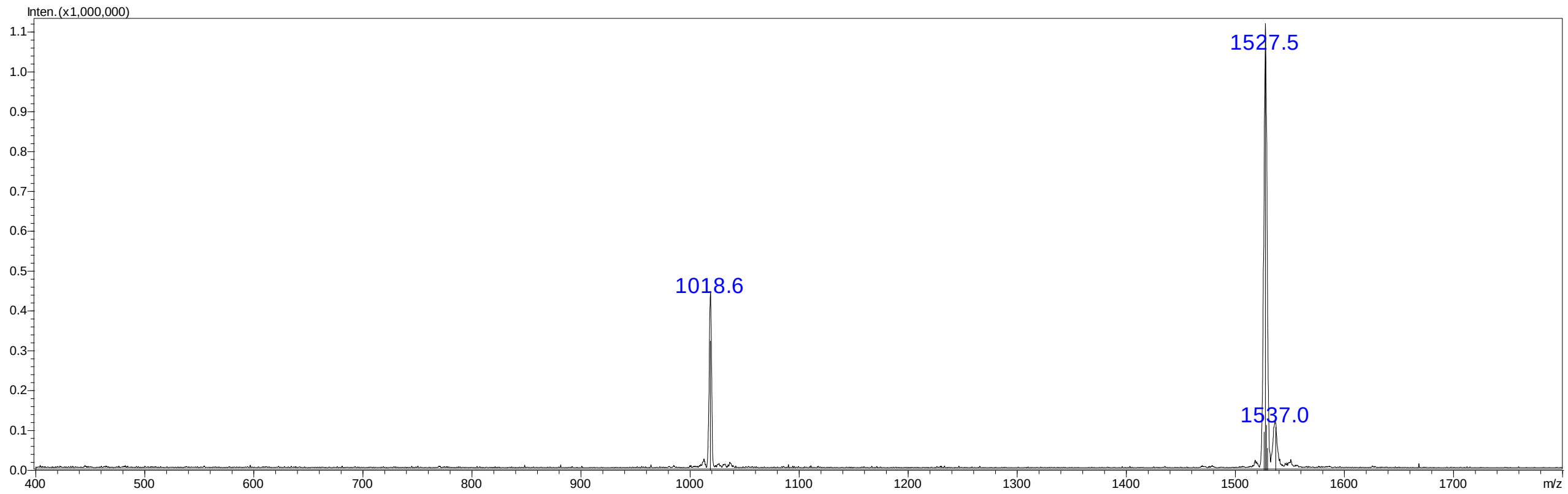

Figure S11. ESI-MS spectrum of cyclic, reduced [C14U,C26U]-kalata B2.

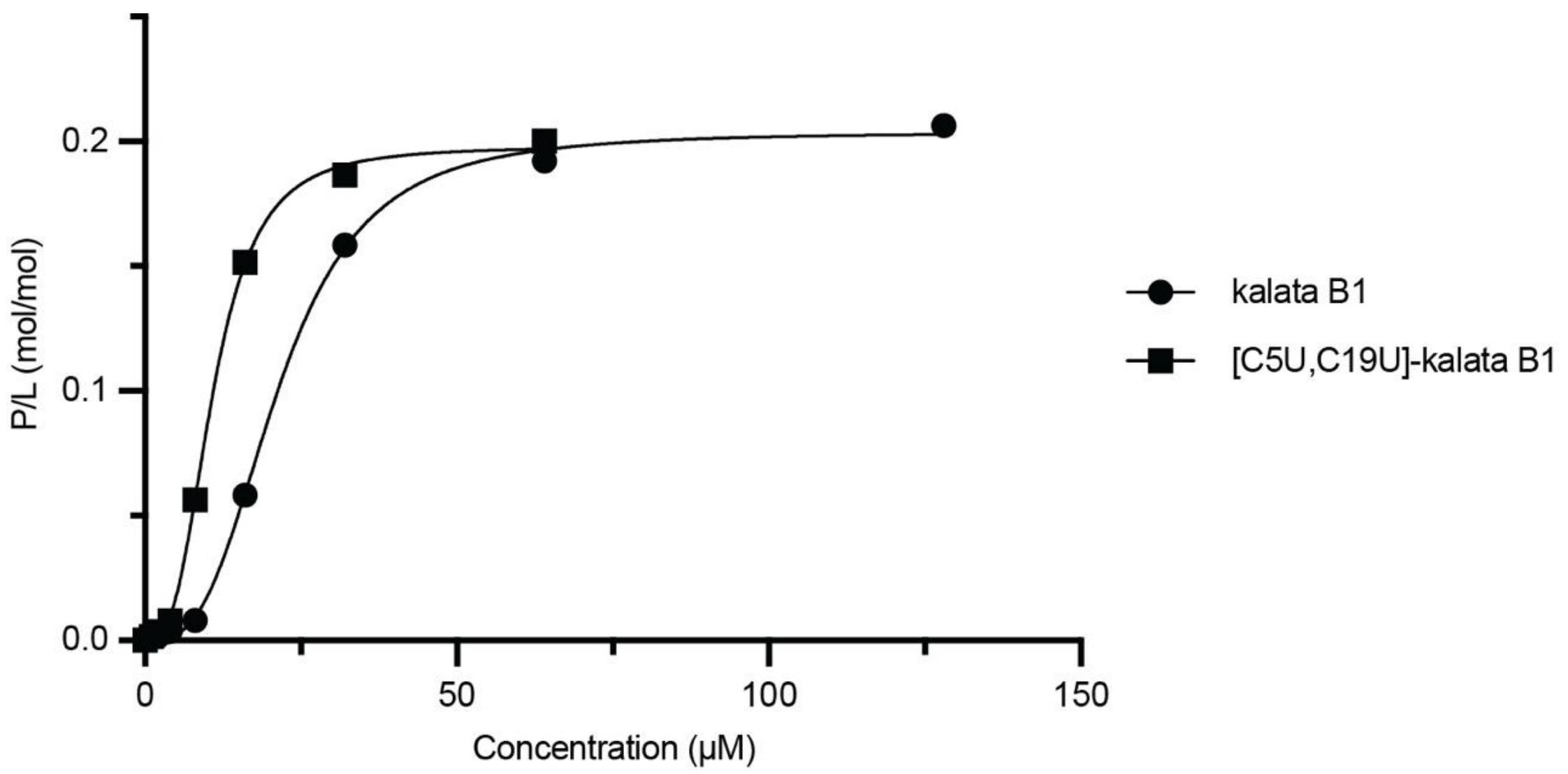

Figure S12. Binding dose-response curve of kalata B1 and [C5U,C19U]-kalata B1 over POPC/POPE (80:20) bilayers onto L1 chip surface monitored by SPR.
